# Understanding the Structure and Dynamics of Nanocellulose-Based Composites with Neutral and Ionic Poly(methacrylate) Derivatives Using Inelastic Neutron Scattering and DFT Calculations

**DOI:** 10.3390/molecules25071689

**Published:** 2020-04-07

**Authors:** Carla Vilela, Carmen S. R. Freire, Catarina Araújo, Svemir Rudić, Armando J. D. Silvestre, Pedro D. Vaz, Paulo J. A. Ribeiro-Claro, Mariela M. Nolasco

**Affiliations:** 1 CICECO—Aveiro Institute of Materials, Department of Chemistry, University of Aveiro, 3810-193 Aveiro, Portugal; cfreire@ua.pt (C.S.R.F.); catarina.araujo@ua.pt (C.A.); armsil@ua.pt (A.J.D.S.); prc@ua.pt (P.J.A.R.-C.); 2 ISIS Neutron & Muon Source, Rutherford Appleton Laboratory, Chilton, Didcot, Oxfordshire OX11 0QX, UK; svemir.rudic@stfc.ac.uk; 3 Champalimaud Foundation, Champalimaud Centre for the Unknown, Lisbon, 1400-038 Lisboa, Portugal; pedro.vaz@fundacaochampalimaud.pt

**Keywords:** bacterial nanocellulose, nanocomposites, poly(2-hydroxyethyl methacrylate), poly(methacroylcholine chloride), poly(methacroylcholine hydroxide), inelastic neutron scattering, DFT calculations

## Abstract

Bacterial nanocellulose (BC)-based composites containing poly(2-hydroxyethyl methacrylate) (PHEMA), poly(methacroylcholine chloride) (PMACC) or poly(methacroylcholine hydroxide) (PMACH) were characterized by inelastic neutron scattering (INS) spectroscopy, combined with DFT (density functional theory) calculations of model systems. A reasonable match between calculated and experimental spectral lines and their intensities was used to support the vibrational assignment of the observed bands and to validate the possible structures. The differences between the spectra of the nanocomposites and the pure precursors indicate that interactions between the components are stronger for the ionic poly(methacrylate) derivatives than for the neutral counterpart. Displaced anions interact differently with cellulose chains, due to the different ability to compete with the O–H···O hydrogen bonds in cellulose. Hence, the INS is an adequate technique to delve deeper into the structure and dynamics of nanocellulose-based composites, confirming that they are true nanocomposite materials instead of simple mixtures of totally independent domains.

## 1. Introduction

A tour through the kaleidoscopic portfolio of materials developed in the last decades, clearly shows that composite materials based on cellulose [1], and bacterial nanocellulose (BC) in particular [2], are quite relevant for myriad domains of applications [3,4,5]. In fact, the exopolysaccharide BC, which is biosynthesized by several non-pathogenic bacteria in the form of membranes with a tree-dimensional network of cellulose nanofibrils [6], has shown potential for drug delivery [7,8,9], wound healing [10,11], bone tissue engineering [12], antimicrobial materials [13], food and food packaging [14,15], water remediation [16,17] and fuel cells [5,18,19], just to mention some fields of application.

In terms of production, BC-based nanocomposites can be prepared by in situ and ex situ methodologies [20], which already enabled the combination of BC with a vast array of synthetic polymers (e.g., polyaniline [21] and Nafion [22]) and biopolymers (e.g., lactoferrin [23] and fucoidan [24]), as well as hybrid materials with metal oxides, metal sulphides and metal nanoparticles [4], and graphene and derivatives [25,26], with the aim of enhancing or adding novel properties to the ensuing materials [2]. Among the available methodologies, the in situ polymerization of monomers with, for instance, (metha)acrylate functional groups, is a simple top-down method that promotes the use of BC without altering its valuable and unique three-dimensional structure [2]. Acrylamide [27], acrylic acid [28], glyceryl monomethacrylate [29], 2-ethoxyethyl methacrylate [29], 2-aminoethyl methacrylate [30], methacryloyloxyethyl phosphate [31], *N*-methacryloyl glycine [7] and bis[2-(methacryloyloxy)ethyl] phosphate [32], are examples of (meth)acrylic monomers that have already been dispersed and polymerized into the BC network, originating materials with the most assorted properties.

The fundamental understanding of the interactions between the individual components in nanocomposites is of paramount importance to withdraw structure-property correlations that can contribute to optimize and modulate the properties of the resulting materials and, thus, surpass the scientific and technological challenges associated with their production. One of the techniques that is gaining momentum to characterize the structure of bulk materials at a molecular level is inelastic neutron scattering (INS) spectroscopy, which provides a unique assessment of the structural dynamics of hydrogenous materials that is not always possible by its optical counterparts, namely infrared and Raman spectroscopy [33]. The vibrational spectroscopy technique that makes use of neutrons to probe the dynamics of atoms and molecules in solids, benefits from the proportionality of the signal intensity to the nuclei displacement and nuclei cross-section, the sensitivity to hydrogen atom vibrations, the truancy of selection rules for the vibrational modes activity, and the coverage of the whole molecular vibrational range including the low-frequency region of the vibrational spectra [33].

The INS spectroscopy can additionally profit from the combination with density functional theory (DFT) calculations. The INS spectrum is a quantitative measurement of the vibrational density of states, and the different contributions to the total spectrum can be computed separately with high accuracy, using DFT. Thus, the experimental INS spectrum can be matched to the calculated spectra of different proposed structures and the structures that do not contribute to the experimental profile can be confidently discarded [34]. This approach, required for amorphous materials and described in detail by Harrelson et al. [34], has been successfully used to infer the local structure of polymers from INS spectra. For instance, it has been used to experimentally assess the random nature of 2,4-poly(ethylene furanoate) [35], to identify the zigzag structure of confined poly(ethylene oxide) [36] and to conclude on the side chains flattening in doped poly(hexylthiophene) [34].

The first studies dealing with the characterization of cellulose by INS were published by Muller and coworkers two decades ago [37,38]. In these studies, the authors were able to identify the bands assigned to O−H and C−H stretching modes, together with the angle bending motions involving hydrogen atoms (1180−1500 cm^−1^), OH out-of-plane modes (550−950 cm^−1^) and C−C and C−O torsional modes (<300 cm^−1^) in different types of cellulose [37]. Moreover, the authors also managed to identify two bands allocated to the stretching modes of intramolecular hydrogen bonds at low frequencies (ca. 120 and 176 cm^−1^) by combining INS with the deuteration of hydroxyl groups in the non-crystalline regions of native cellulose [38]. Further down the road, Araujo et al. [39] explored the dynamics of hydrogen bonds in cellulose samples from distinct sources, namely microcrystalline cellulose, cotton cellulose, kraft pulp cellulose and BC, by INS (with the help of the TOSCA spectrometer) [40,41,42,43] and were able to easily distinguish between the crystalline forms of I_α_ and I_β_ allomorphs in the 700−800 cm^−1^ region. Moreover, this study set the stage for the future use of this technique to characterize BC-based materials, and in particular of nanocomposites [39].

Under these premises and following our interest in the design and characterization of advanced and multifunctional BC-based nanocomposites [11,13,24], the present work intends to study the dynamics of hydrogen-bond networks in BC-based nanocomposites containing poly(2-hydroxyethyl methacrylate) (PHEMA), poly(methacroylcholine chloride) (PMACC) and poly(methacroylcholine hydroxide) (PMACH) by INS spectroscopy. These nanocomposites, previously developed by our research group [44,45], were selected as a good basis for BC-based nanocomposite materials with neutral and ionic functional groups, viz. hydroxyl and quaternary ammonium moieties, respectively. The characterization of the nanoscale structure of these nanocomposites by INS spectroscopy will allow a sound interpretation of conformational motifs across the distinct components in the ensuing nanocomposite materials. Moreover, emphasis will be placed on the low frequency modes and hydroxyl torsional motions, i.e., vibrational modes not accessible by conventional vibrational spectroscopic techniques, which will provide information on the packing of the BC-based nanomaterials and intermolecular interactions between the polymeric chains.

## 2. Results and Discussion

**Figure 1:**
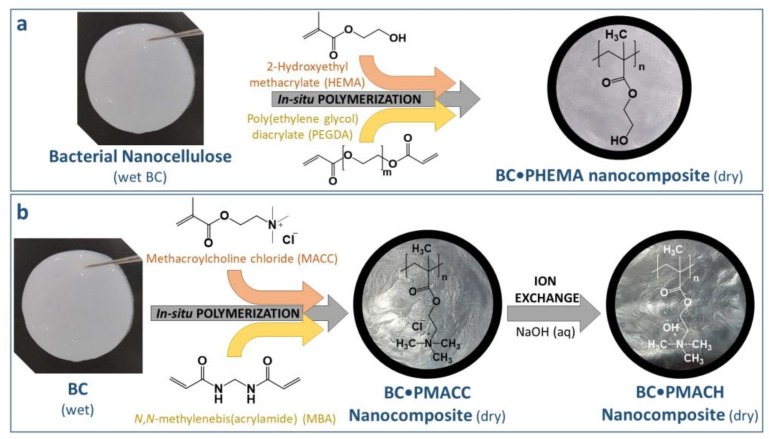
Scheme of the preparation of the bacterial nanocellulose (BC)-based nanocomposites containing neutral (**a**) and ionic (**b**) moieties (evidencing the position of the methyl groups).

In the present study, BC•PHEMA and BC•PMACC nanocomposites with two different polymer contents (Table 1) were prepared by the simple in-situ free radical polymerization of the respective monomers, namely HEMA and MACC (Figure 1), within the BC porous network, according to the procedures reported in our previous studies [44,45]. The polymerization reactions were carried under ecofriendly reaction conditions (i.e., water as solvent and low reaction temperature) and in the presence of a cross-linker (PEGDA and MBA, respectively (Figure 1)), to avoid leaching of the water-soluble polymers (i.e., PHEMA and PMACC (Figure 1)) out of the nanocomposites when in contact with water or aqueous solutions. Additionally, the dried BC•PMACC nanocomposite containing the chloride anion was converted into the basic form via ionic exchange, which yielded the BC•PMACH nanocomposite with the hydroxide anion (Figure 1b). Cross-linked homopolymers of PHEMA and PMACC were also prepared in the absence of BC for comparison purposes. These polymers were selected because of their similar structure that only differs in the ending groups of the side-chains, viz. a neutral hydroxyl moiety in the case of PHEMA and an ionic moiety (quaternary ammonium cation) in the case of PMACC, as depicted in Figure 1.

The structures of pure BC, cross-linked homopolymers (PHEMA and PMACC), BC•PHEMA, BC•PMACC and BC•PMACH nanocomposites were confirmed by solid-state ^13^C CP/MAS NMR, as given in detail in the experimental section, and concur with the data reported in the literature [44,45]. ATR-FTIR and FT-Raman spectra of each material were also obtained in order to check the conformity with previously reported data, as discussed below. Furthermore, BC•PHEMA and BC•PMACC nanocomposite materials, together with the corresponding individual components, were analyzed by INS spectroscopy and the assignments were based on the spectra obtained from Gaussian09 calculations. Particular focus was placed on the vibrational modes that are not accessible by conventional vibrational spectroscopic techniques, namely the low frequency modes and hydroxyl torsional motions of BC, which provide information on the packing of the BC-based nanomaterials and intermolecular interactions between polymer chains, respectively, as discussed in detail in the following sections.

### 2.1. INS Spectrum of Pure Compounds

**Figure 2:**
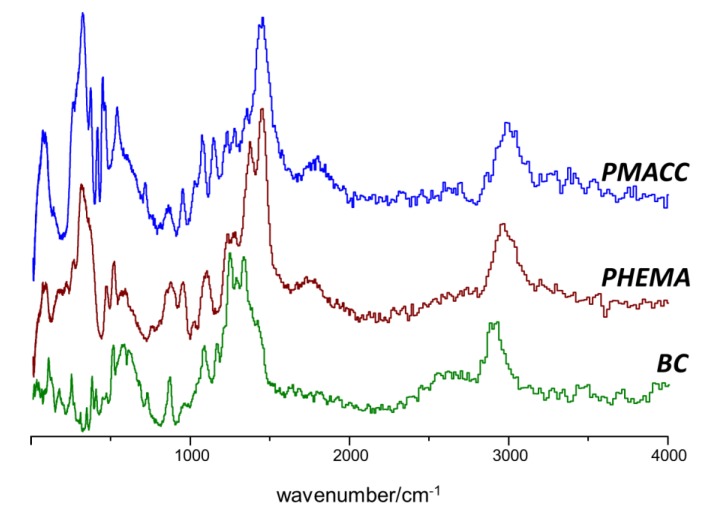
Inelastic neutron scattering (INS) spectra of PMACC (top), PHEMA (middle) and BC (bottom) in the 20–4000 cm^−1^ range (arbitrary intensity units).

Figure 2 presents the INS spectrum of the cross-linked polymers, PHEMA and PMACC, and dry BC. The INS spectrum of dry BC has been the subject of detailed description and assignment in a previous study [39]. Full assignments of the INS spectra of PMACC and PHEMA, presented in Tables S1 and S2 (Supplementary Material), were herein obtained from quantum chemical calculations with model compounds (triads). Although the search of the full configurational landscape was not intended in this work, isotactic, syndiotactic and heterotactic triads were used to assist the assignment of vibrational spectra.

The predicted spectra for these model triads have been compared with INS, infrared and Raman spectra. Comparisons with the infrared spectra (Figures S1 and S2, Supplementary Material) show that a crude description of the experimental spectrum of PMACC was obtained from the three forms, although the best fit was obtained from the isotactic model. These results, although impaired by model limitations, suggest that PMACC had, at least, large isotactic domains, and the isotactic triad was used in simulations thereof. In the case of PHEMA, the isotactic model failed to describe the spectrum, namely in what concerns the general intensity profile in the region of C–C stretching modes (ca. 1100–1200 cm^−1^). The same general conclusions could be drawn for the Raman spectra for both PMACC and PHEMA (Figure S3, Supplementary Material). Since syndiotactic and heterotactic forms provide a reasonable description of the experimental spectra of PHEMA, this homopolymer was assumed to be atactic and an average spectrum of the two triads was used for INS simulation.

**Figure 3:**
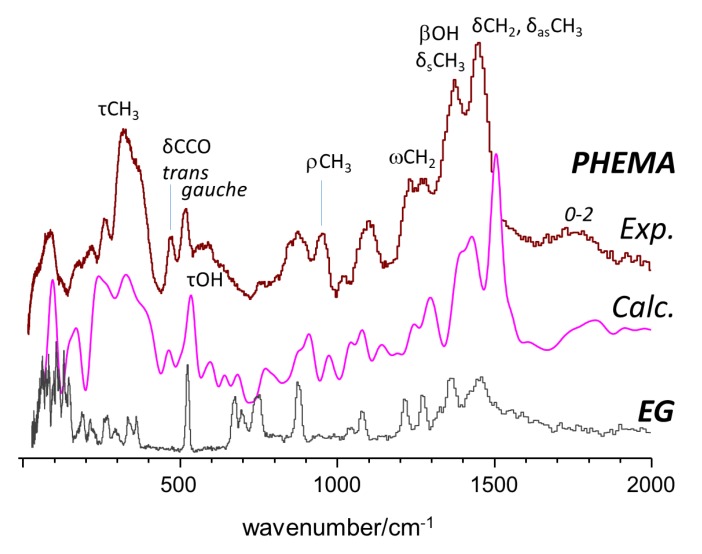
INS spectra of PHEMA in the 20–2000 cm^−1^ range (top), compared with the calculated spectra generated from identical contributions of heterotactic and syndiotactic triads (middle), and the INS spectrum of ethylene glycol (bottom) [36] (arbitrary intensity units).

The INS spectrum of PHEMA is shown in Figure 3 (top). The most prominent features, related with the O–CH_2_–CH_2_–OH side fragment, were easily identified, as they could be directly derived from the INS spectrum of ethylene glycol (Figure 3, bottom) [36]. For instance, the band pair at ca. 475/520 cm^−1^ provided a measure of the *trans*/*gauche* population, indicating that both configurations were present in the polymer structure. The strong signal of methyl torsions was observed at ca. 300–400 cm^−1^, in the shape of a broad band with two major components, indicating the presence of two types of methyl groups. In the model calculations (Figure 3, middle), the methyl groups with the highest torsional frequency were those involved in C–H···O contacts with a neighboring side chain.

**Figure 4:**
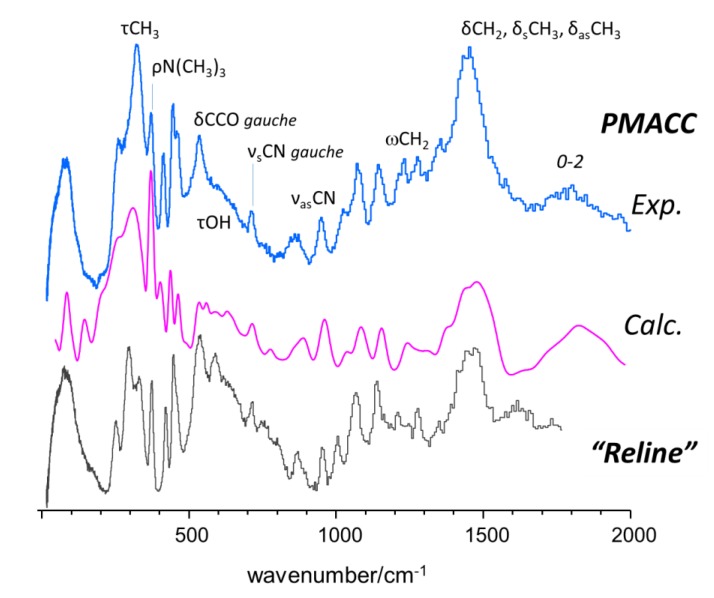
INS spectra of PMACC in the 20–2000 cm^−1^ range (top), compared with the calculated spectra from the isotactic triad (middle), and the INS spectrum of reline (bottom) [46] (arbitrary intensity units).

The calculated INS spectrum of PMACC (Figure 4, middle) provided a good qualitative description of the experimental spectrum (Figure 4, top). It should be mentioned that the INS spectrum of PMACC was mostly dominated by the vibrations of the choline chloride fragment, and presented a remarkable similarity with the INS spectrum of “reline” (choline chloride and urea eutectic mixture) [46] shown in Figure 4 (bottom). This similarity provided further reliance on the interpretation of spectral features, as discussed below. In particular, the broad profile of the strong bands resulting from methyl groups torsions, at ca. 300 cm^−1^, is a clear indication of a non-crystalline ambience of the trimethylammonium groups.

### 2.2. Interactions between Components in the Nanocomposites

**Figure 5:**
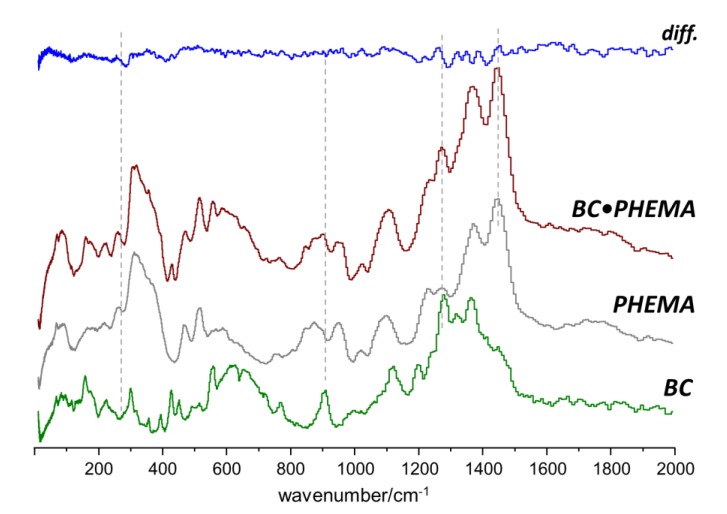
INS spectra of BC•PHEMA nanocomposite, PHEMA and BC in the 20–2000 cm^−1^ range. The top line is the difference between nanocomposite spectrum (BC•PHEMA) and the weighted sum of pure components spectra (PHEMA + BC).

**Figure 6:**
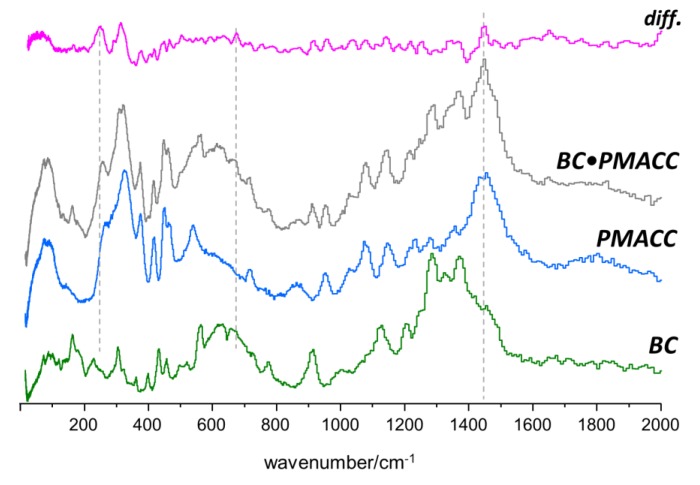
INS spectra of BC•PMACC nanocomposite, PMACC, and BC in the 20–2000 cm^−1^ range. The top line is the difference between nanocomposite spectrum (BC•PMACC) and the weighted sum of pure components spectra (PMACC + BC).

**Figure 7:**
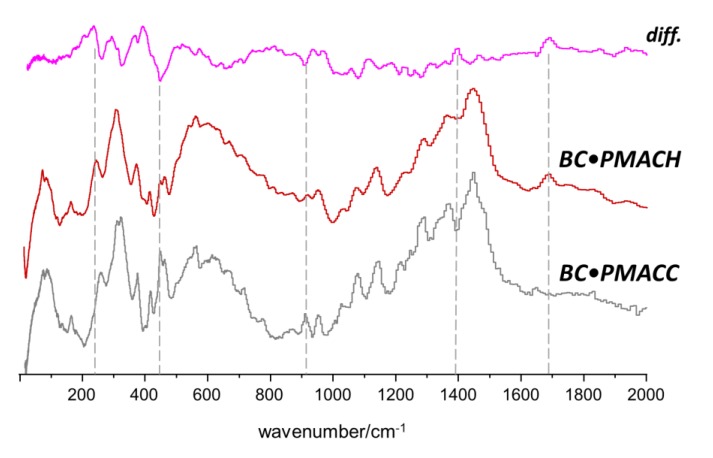
INS spectra of BC•PMACH and BC•PMACC nanocomposites in the 20–2000 cm^−1^ range. The top line is the difference between BC•PMACH and BC•PMACC spectra.

The INS results obtained for the BC•PHEMA, BC•PMACC and BC•PMACH nanocomposites are summarized in Figure 5, Figure 6 and Figure 7, respectively. The top lines in each figure are the differences between spectra and emphasize the spectral changes promoted by the formation of the nanocomposite.

A general conclusion that can be drawn from the three systems is that, although there was strong evidence of new interactions resulting from the nanocomposite formation and the consequent interaction between the two phases, the preservation of BC morphology/ultrastructure was also clearly demonstrated. In fact, the INS spectra of the nanocomposites exhibited several features that were not compatible with the presence of independent BC and poly(methacrylate) domains, i.e., must arise from the interactions between components in the nanocomposites. On the other hand, the region ascribed to the large amplitude collective modes (ca. < 150 cm^−1^) did not evidence the large changes expected for a significant ultrastructural modification of the BC structure or of the poly(methacrylate). This clearly suggests that the methacrylate polymerization occurs within a non-constrained space and BC fibrils keep their wide-net structure. The results were consistent with nanocomposite materials made of entangled chains of a synthetic cross-linked polymer and a fibrillar biopolymer, with intermolecular interactions between polymer fragments in multiple contact points.

In the case of BC•PHEMA nanocomposites, the stronger interactions between components were expected to be those arising from the presence of hydroxyl groups in both chains and the additional hydrogen-bond acceptors (carboxyl oxygen atoms) in PHEMA. The difference spectra (nanocomposite minus pure components, Figure 5) evidenced the very small differences promoted by BC-PHEMA interactions. These small differences did not result from subtraction artifacts, as they were consistently observed for both BC•PHEMA samples with distinct compositions.

It has been shown by several authors [38,47,48,49] that cellulose–water interactions occurred preferentially through the primary hydroxyl groups and had a strong effect on the INS band at ca. 910 cm^−1^ (CH_2_ rocking mode) [39]. If similar interactions occur between PHEMA hydroxyl groups and BC, a similar band change would be observed. However, the region of the CH_2_ rocking mode corresponded to a nearly flat line in the difference spectrum, thus discarding significant interactions of this kind.

In fact, the comparisons between the BC•PHEMA samples with two different contents of PHEMA, viz. 63% ± 2% and 86% ± 4%, indicate that the INS spectrum of BC remained mostly unaffected in the nanocomposite, and the observed differences should be ascribed to the PHEMA component. These include the intensity transfer at ca. 250 cm^−1^ and several intensity changes in the 1250–1450 cm^−1^ range. While the former may be ascribed to skeletal modes of the polymer, the later are most easily related with conformational-dependent vibrational modes of the CH_2_CH_2_–OH side chain, namely CH_2_ wagging, twisting and scissoring [36].

As for the ionic nanocomposites (BC•PMACC and BC•PMACH, Figure 6 and Figure 7, respectively), the INS spectra revealed a larger disturbance in the structure of the pure samples. The interaction between the components was stronger than for the non-ionic nanocomposite, an effect that must stem from the mobility of the anions. In the case of BC•PMACC, the difference spectrum (Figure 6, top line) clearly presented two new well-defined bands in the region of the methyl torsions (ca. 200–350 cm^−1^). In addition, it displays intensity changes coinciding with several bands of both BC and PMACC for the whole region shown in Figure 6. The rugged profile of the difference spectrum in the ca. 500–1200 cm^−1^ region contrasted with the nearly flat line observed for BC•PHEMA in the same region. Difference maxima and minima coincided with position of bands from both PMACC and BC, and this was assumed as evidence that both components of the nanocomposite were disturbed from their pure sample ultrastructure. The exchange of chloride by hydroxide anions in PMACH strengthened the effects in the region of methyl torsions and added several intensity changes along the spectrum. The most relevant regions are highlighted in Figure 7.

The well-defined bands emerging in the 200–350 cm^−1^ region can be reasonably assigned to the torsional motions of the methyl groups of methacroylcholine moieties. In a previous INS report [46], it was observed that methyl torsions of choline chloride are good probes of the proximity of chloride ions relative to the methyl groups. In the crystal, chloride ions are hydrogen-bonded to the methyl groups and the methyl torsional motions are observed as sharp bands in the 286–349 cm^−1^ interval. When the crystalline structure is disrupted and chloride ions are partially displaced from methyl groups, torsions became less hindered, and a corresponding downshift to the 252–333 cm^−1^ range is observed [46]. In the cross-linked PMACC (Figure 6, middle), the broad profile of methyl torsions is characteristic of its amorphous nature, with significant methyl-chloride interactions. The emergence of the two new bands at lower frequency in BC•PMACC (Figure 7, bottom) and BC•PMACH (Figure 7, middle) was thus ascribed to the torsions of free (or nearly free) methyl groups in the nanocomposite, resulting from the displacement of the anions towards the cellulose fibrils. Since the effect was strengthened by the chloride-to-hydroxide exchange (Figure 7), it may be concluded that fewer hydroxide anions are bound to the trimethylammonium heads of the synthetic polymer, giving preference to the interactions with the cellulose fibrils. This is also evident in the higher wavenumber regions, as discussed below.

**Figure 8:**
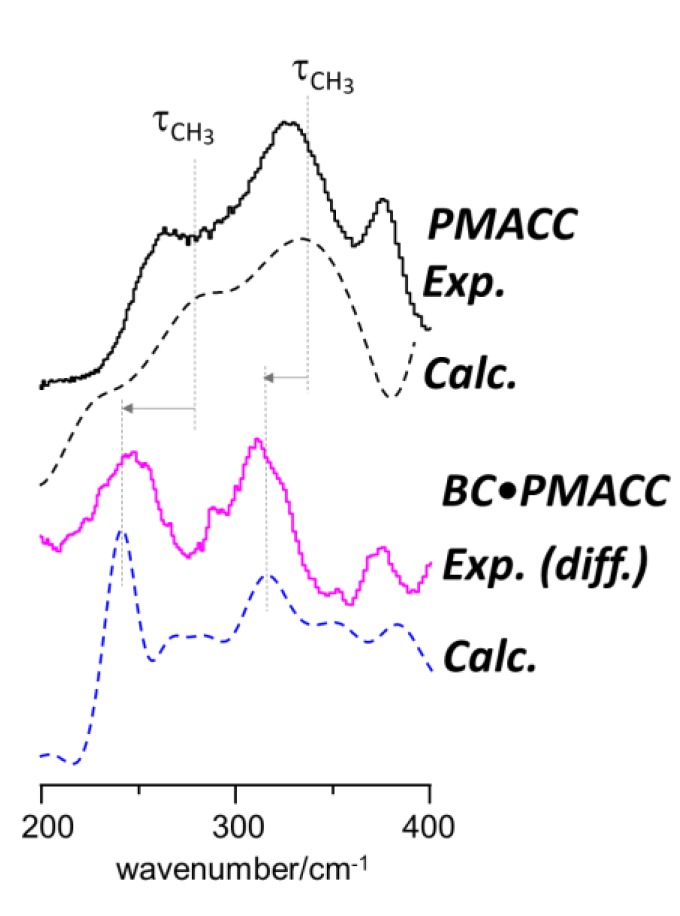
Observed (full lines) and calculated (dashed lines) INS profiles for the methyl torsions in PMACC. Top lines are an insight of Figure 4, and compare the experimental INS spectra of PMACC with the with the calculated spectrum for the methyl groups bound to chloride ions in the isotactic triad; bottom lines compare the INS difference spectra of BC•PMACC with the calculated profile for free methyl groups (no chloride ions) in the isotactic triad.

As stated before, the contributions to the INS experimental intensities can be efficiently evaluated from DFT calculations. A reasonable match between calculated and experimental intensities can be used to validate a possible structure, while a mismatch justifies its exclusion. The assignment of the emerging bands in the 200–350 cm^−1^ region to methyl group torsions, as discussed above, is clearly supported by DFT calculations, whose results are summarized in Figure 8. Figure 8 compares the INS frequencies and intensities of the methyl torsions for the PMACC triads with and without chloride ions bound to the trimethylammonium heads with the experimental INS spectra of PMACC and BC•PMACC, respectively. As it can be seen on Figure 8, the intensity maxima obtained with aCLIMAX software are in excellent agreement with the observed bands. Of course, the perfect match between calculated and observed maxima in bottom lines of Figure 8 (difference spectrum and calculated spectrum for the isotactic triad without chloride anions) may be circumstantial, but it is still important to highlight that calculations predict the emergence of new bands for methyl torsions, at lower wavenumbers, as a result of the displacement of chloride ions.

The remaining changes observed in the difference spectra of Figure 6 and Figure 7 where analyzed and assigned in a similar way, considering PMACC triads and glucose dimers (cellobiose type structure), with and without interactions with the chloride and hydroxide anions.

**Figure 9:**
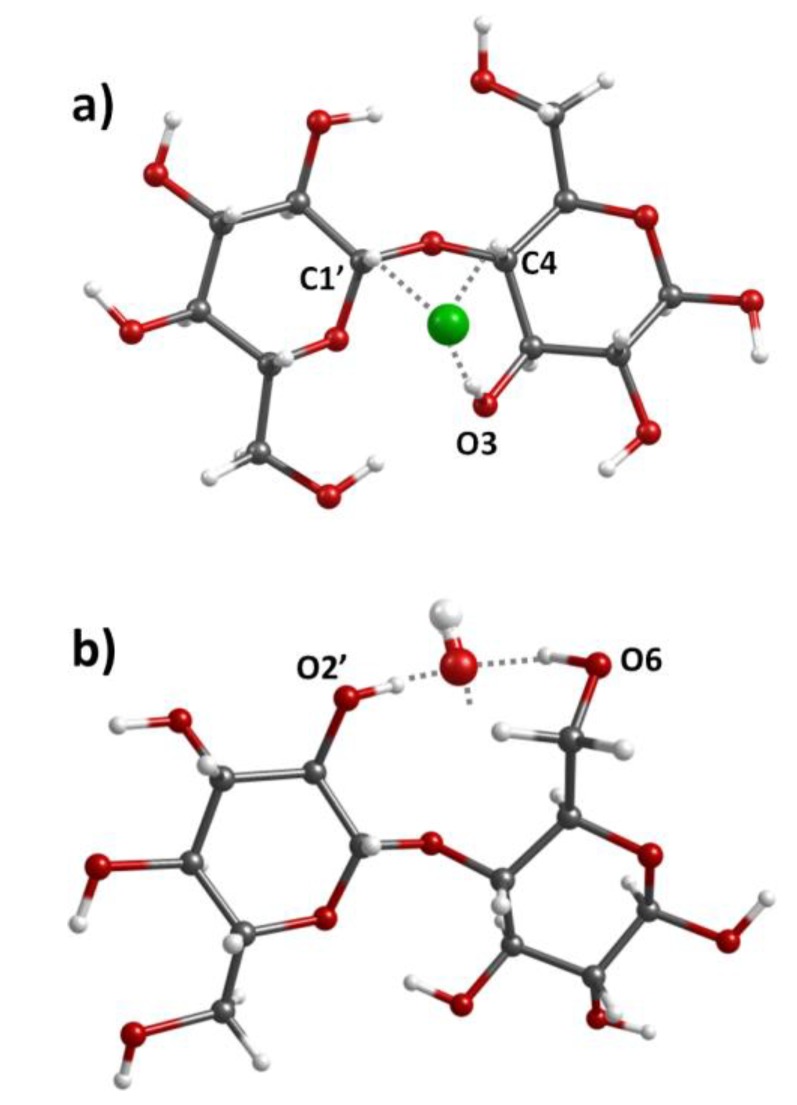
(**a**) Lowest energy optimized structure of the cellobiose-chloride model. The contacts are O(3)–H···Cl (205 pm), C(4)–H···Cl (260 pm) and C(1’)–H···Cl (266 pm) and (**b**) example of the cellobiose-hydroxide structure, with preference for O–H···O contacts. For reasons of readability, the molecule of the third hydroxyl group is omitted.

In BC•PMACC, a relevant intensity transfer was observed in the C–H bending region, with an intensity loss at ca. 1390 cm^−1^ and a corresponding intensity gain ca. 1445 cm^−1^ (see Figure 6). DFT calculations show that the intensity transfer from lower to higher wavenumber cannot be associated with the chloride displacement in PMACC. Instead, it can be explained by the interactions of chloride ions with cellulose units, which occurred through multiple C–H···Cl and O–H···Cl contacts. The C–H···Cl contacts predominated over O–H···Cl ones, because O–H groups were already engaged in strong hydrogen bonds. For the lowest energy structure, the cellobiose-chloride model, shown in Figure 9a, the C–H···Cl contacts promoted a blue-shift of the antisymmetric C1’/C4–H bending of ca. 45 cm^−1^, as observed in the experimental INS spectrum.

The chloride-by-hydroxide replacement in BC•PMACH seems to reverse this effect in the CH bending region (Figure 7), while promoting several other intensity changes, including the rising of a new band at ca. 1690 cm^−1^. These observations indicate that chloride and hydroxide anions have a different interaction with the cellulose chains. In fact, DFT calculations with the cellobiose-hydroxide model point to the formation of tetrahedral hydration-type interactions [50] between hydroxide and the hydroxyl groups (Figure 9b). According to calculations, these interactions explain the reversal of the shift in C–H bending region, and contribute to the observed intensity changes in the region of the 910 cm^−1^ band (CH_2_ rocking mode, sensitive to conformation of glucose primary hydroxyl group) and to the emerging of the 1690 cm^−1^ band (due to O–H bending modes of the new O–H···OH^−^ moiety).

## 3. Materials and Methods

### 3.1. Chemicals and Materials

2,2-Azobis(2-methylpropionamidine) dihydrochloride (AAPH, 97%), 2-hydroxyethyl methacrylate (HEMA, 97%, stabilized), potassium persulfate (KPS, ≥99%), methacroylcholine chloride solution (MACC, 80 wt% in H_2_O), *N*,*N*-methylenebis(acrylamide) (MBA, 99%) and poly(ethylene glycol) diacrylate (PEGDA, Mn 250) were purchased from Sigma-Aldrich (Sintra, Portugal) and used as received without any further purification. Other chemicals and solvents were of laboratory grade.

### 3.2. Nanocellulose Samples

Bacterial nanocellulose (BC) membranes, with ca. 99 wt% water content, were produced in our laboratory using the bacterial strain *Gluconacetobacter sacchari* following a procedure described elsewhere [51]. The wet membranes were dried at 60 °C in a vacuum drying oven (Thermo Fisher Scientific, Massachusetts, USA) until ca. 5 wt% water content. ^13^C CP/MAS NMR (BC): δ 65.2 ppm (C6), 71.4–74.3 ppm (C2,3,5), 90.0 ppm (C4) and 104.8 ppm (C1).

### 3.3. Synthesis of Cross-Linked Polymers

The cross-linked PHEMA was synthesized via the free radical polymerization of HEMA (2.0 g, 15.4 mmol) in the presence of PEGDA (100 mg, 5% *w*/*w* relative to monomer), KPS (20 mg, 1% *w*/*w* relative to monomer) and 5 mL of water. The reaction mixture was left at 70 °C for 6 h under inert atmosphere [44]. The gel-like material was washed with water, freeze-dried and a white powder was obtained. ^13^C CP/MAS NMR (PHEMA): δ 18.5 ppm (CH_3_ of polymer backbone), 45.1 ppm (quaternary C of polymer backbone), 55.4 ppm (CH_2_ of polymer backbone), 60.0 ppm (OCH_2_), 67.3 ppm (CH_2_HO) and 178.1 ppm (C=O).

The cross-linked PMACC was synthesized by the free radical polymerization of MACC (2.0 g, 9.6 mmol) in the presence of MBA (100 mg, 5% *w*/*w* relative to monomer), AAPH (20 mg, 1% *w*/*w* relative to monomer) and 5 mL of water [45]. The polymer was freeze-dried and obtained in the form of a white solid. ^13^C CP/MAS NMR (PMACC): δ 18.9 ppm (CH_3_ of polymer backbone), 45.1 ppm (quaternary C of polymer backbone), 54.5 ppm (N^+^(CH_3_)_3_ and CH_2_ of polymer backbone), 59.2 ppm (OCH_2_), 65.1 ppm (CH_2_N^+^(CH_3_)_3_) and 177.8 ppm (C=O).

### 3.4. Preparation of BC-Based Nanocomposites

Wet BC pellicles (ca. 400 mg, ca. 7 cm diameter) with 40% water were placed in stoppered glass-reactors containing an aqueous solution of monomer (HEMA or MACC), cross-linker (PEGDA or MBA, respectively, 5.0% *w*/*w* relative to monomer) and radical initiator (KPS or AAPH, respectively, 1.0% *w*/*w* relative to monomer), according to the compositions listed in Table 1. The glass-reactors with the reaction mixtures were purged with nitrogen and placed in an oil bath at 70 °C for 6 h. Afterwards, the nanocomposites were repeatedly washed with distilled water, oven dried at 40 °C for 12 h, and kept in a desiccator. All experiments were made in quintuplicate.

The BC•PMACH nanocomposites were prepared by converting the dried BC•PMACC nanocomposite into the basic form via ionic exchanging with an aqueous solution of 1 M NaOH for 24 h at room temperature [45]. The basic nanocomposites were again washed repetitively with distilled water, dried at 40 °C for 12 h and maintained in desiccators until their use.

^13^C CP/MAS NMR (BC•PHEMA): δ 18.5 ppm (CH_3_ of polymer backbone), 45.1 ppm (quaternary C of polymer backbone), 55.4 ppm (CH_2_ of polymer backbone), 60.0 ppm (OCH_2_), 65.2 ppm (C6 of cellulose), 67.3 ppm (CH_2_HO), 71.4–74.3 ppm (C2,3,5 of cellulose), 90.0 ppm (C4 of cellulose), 104.8 ppm (C1 of cellulose) and 178.1 ppm (C=O).

^13^C CP/MAS NMR (BC•PMACC and BC•PMACH): δ 18.9 ppm (CH_3_ of polymer backbone), 45.1 ppm (quaternary C of polymer backbone), 54.5 ppm (N^+^(CH_3_)_3_ and CH_2_ of polymer backbone), 59.2 ppm (OCH_2_), 65.1 ppm (CH_2_N^+^(CH_3_)_3_), 65.2 ppm (C6 of cellulose), 71.4–74.3 ppm (C2,3,5 of cellulose), 90.0 ppm (C4 of cellulose), 104.8 ppm (C1 of cellulose) and 177.8 ppm (C=O).

### 3.5. Solid-State Carbon Cross-Polarization/Magic-Angle-Spinning Nuclear Magnetic Resonance (^13^C CP/MAS NMR)

Spectra were collected on a Bruker Avance III 400 spectrometer (Bruker Corporation, Massachusetts, USA) operating at a B0 field of 9.4 T using 12 kHz MAS with proton 90° pulse of 3 µs, time between scans of 3 s and a contact time of 2000 µs. ^13^C chemical shifts were referenced to glycine (C=O at δ 176 ppm).

### 3.6. Attenuated Total Reflection-Fourier Transform Infrared (ATR-FTIR)

Spectra were recorded on a Bruker Tensor 27 spectrometer (Bruker Corporation, Massachusetts, USA), using a Golden Gate single reflection diamond ATR system, with no need for sample preparation. All spectra are the average of two counts of 128 scans each, with 2 cm^−1^ resolution.

### 3.7. Room-Temperature Fourier Transform Raman (FT-Raman)

Spectra were recorded on an RFS-100 Bruker FT spectrometer (Bruker Corporation, Billerica, MA, USA), using an Nd:YAG laser with an excitation wavelength of 1064 nm. All spectra are the average of three repeated measurements of 150 scans each, with 2 cm^−1^ resolution.

### 3.8. Neutron Scattering Experiments

Inelastic neutron scattering experiments were performed with the TOSCA spectrometer [42,43], an indirect geometry time-of-flight spectrometer at the ISIS Neutron and Muon Source at the Rutherford Appleton Laboratory (Chilton, UK) [52]. The final neutron energy was approximately 3.95 meV, and the energy resolution, ΔE/E was about 1.25% between 400 and 1600 cm^−1^ and increased to more than 1.5% in 2400–4000 cm^−1^ range.

Each sample, with a total amount of 0.8–1.5 g, was packed inside a flat thin-walled aluminum can of 4.8 cm height and 4 cm width, with a path length of 2 mm, which were then mounted perpendicular to the beam using a regular TOSCA centre-stick. Samples were “shock-frozen” by quenching in liquid nitrogen before placement in the beam path. This procedure (“shock-freezing”) aims at preserving, as far as possible, the morphology of amorphous regions at the low temperature required by experiment, while it is not expected to disturb the structure of cellulose nanofibrils. Spectra were collected below 20 K and the data collection time was typically 5–8 h.

### 3.9. Spectral Analysis

In order to emphasize the spectral differences resulting from the formation of the nanocomposites, the spectra of pure components were subtracted from the spectrum of each nanocomposite sample. The procedure requires an adequate weighting of pure components spectra to match the nanocomposite composition. In the case of the INS spectra, the resulting difference spectra revealed the presence of bands belonging to the aluminum can. The contribution from the aluminum scattering to each spectrum was negligible (for the sample amounts used here) but its magnitude depended on the sample/aluminum ratio, so the difference spectra was sensitive to slightly different contributions in each spectrum. These aluminum bands in the difference spectra were removed by subtracting the INS spectrum of the empty aluminum can.

### 3.10. Quantum Chemical Calculations

Discrete quantum chemical calculations for HEMA and MACC triads (isotactic, syndiotactic and heterotactic) and for BC-chloride and BC-hydroxide complexes were computed using the Gaussian 09 software [53]. Optimized geometries and vibrational frequencies were obtained with the M062X functional and the 6-311+G(d) basis set, using the Int=FineGrid option, as implemented in Gaussian. The M06-2X/6-311+G*level was considered as it provides a good compromise between accuracy and computational costs. The M06-2X functional has been proven to have better performance than the most popular B3LYP for main-group structures and noncovalent interactions [54,55,56,57].

All the optimized structures were found to be real minima, with no imaginary frequencies. The atomic displacements in each mode, that are part of the Gaussian 09 output, enable visualization of the modes to aid assignments and are also all that is required to generate the INS spectrum using aCLIMAX software [58]. aCLIMAX calculates INS intensities incorporating multi-quanta transitions and instrumental bandwidth, producing a calculated spectrum that is easily compared with the experimental spectrum.

## 4. Conclusions

INS spectroscopy, combined with DFT calculations on model systems, was used to characterize BC-based nanocomposites containing neutral and ionic polymeric matrices. The results highlight the use of inelastic neutron spectroscopy, combined with ab initio calculations on model compounds, as an appropriate tool to infer structural motifs of disordered or amorphous materials. A reasonable match between calculated and experimental intensities was used to support the vibrational assignment of the observed bands and to validate the possible structures. Model calculations for the pure cross-linked polymeric matrices suggest that the ionic PMACC had, at least, large isotactic domains, while the neutral PHEMA was assumed to be atactic, since both syndiotactic and heterotactic forms provided a reasonable description of its experimental spectra.

The INS spectra revealed that BC and the poly(methacrylate) derivatives form true composites (not a mixture of totally independent domains), in which the valuable three-dimensional structure of BC was preserved. The interaction between the components was stronger for ionic than for the neutral nanocomposites, an effect attributed to the mobility of the anions. The emergence of the two new bands at lower frequency in BC•PMACC and BC•PMACH was ascribed to the torsions of free (or nearly free) methyl groups in the nanocomposite, resulting from the displacement of the anions towards the cellulose fibrils. The observed spectra clearly show that chloride and hydroxide anions had different interactions with BC. The chloride anion had a limited ability to compete with the cellulose O-H···O bonds and tended to interact with the cellulose fibrils surface through multiple C–H···Cl contacts. On the other hand, the hydroxide anion had a more disruptive interaction with the O-H groups of cellulose fibrils, probably forming tetrahedral hydration-type structures.

## Figures and Tables

**Table 1:** List of the prepared nanocomposites with the respective weight compositions.

Nanocomposites	Nominal Composition	Measured Composition	
	*W*_BC_/*W*_monomer_	*W*_polymer_/*W*_total_	*W*_BC_/*W*_total_
BC•PHEMA	1:2	0.63 ± 0.02	0.37 ± 0.02
	1:4	0.86 ± 0.04	0.14 ± 0.04
BC•PMACC	1:2	0.42 ± 0.06	0.58 ± 0.06
	1:4	0.70 ± 0.05	0.30 ± 0.05
BC•PMACH	1:4	0.70 ± 0.05	0.30 ± 0.05

## Supplementary Materials

The following are available online at https://www.mdpi.com/1420-3049/25/7/1689/s1, Figure S1: Infrared spectra of PHEMA in the 400–1900 cm^−1^ range (a), compared with the calculated spectra for syndiotactic (b), heterotactic (c) and isotactic (d) triads. Figure S2: Infrared spectra of PMACC in the 400–1900 cm^−1^ range (a), compared with the calculated spectra for isotactic (b), syndiotactic (c) and heterotactic (d) triads. Figure S3: Raman spectra of PHEMA in the 100–1900 cm^−1^ range (a), compared with the calculated spectra for syndiotactic (b), heterotactic (c) and isotactic (d) triads. Table S1: Assignment of INS spectra of PMACC. Table S2: Assignment of INS spectra of PHEMA.
