# Supplementary material for: Understanding the Structure and Dynamics of Nanocellulose-Based Composites with Neutral and Ionic Poly(methacrylate) Derivatives Using Inelastic Neutron Scattering and DFT Calculations
Source: Molecules. 2020 Apr 7;25(7):1689. doi: 10.3390/molecules25071689 (PMC7180936; doi:10.3390/molecules25071689)
Supplement: Supplementary file 1 [file molecules-25-01689-s001.pdf]

Supplementary Material

Understanding the structure and dynamics of nanocellulose-based composites with neutral and ionic poly(methacrylate) derivatives using inelastic neutron scattering and DFT calculations

Carla Vilela [1,*], Carmen S. R. Freire [1], Catarina Araújo [1], Svemir Rudić [2], Armando J. D. Silvestre [1], Pedro D. Vaz [3] and Paulo J. A. Ribeiro-Claro [1], Mariela M. Nolasco [1,*]

[1] CICECO – Aveiro Institute of Materials, Department of Chemistry, University of Aveiro, 3810-193 Aveiro, Portugal; cfreire@ua.pt (C.S.R.F.); catarina.araujo@ua.pt (C.A.); armsil@ua.pt (A.J.D.S.); prc@ua.pt (P.J.A.R.C.)

[2] ISIS Neutron & Muon Source, Rutherford Appleton Laboratory, Chilton, Didcot, Oxfordshire OX11 0QX, UK; svemir.rudic@stfc.ac.uk (S.R.)

[3] Champalimaud Foundation, Champalimaud Centre for the Unknown, Lisbon, 1400-038 Lisboa, Portugal; pedro.vaz@fundacaochampalimaud.pt (P.D.V.)

* Correspondence: cvilela@ua.pt (C.V.); mnolasco@ua.pt (M.M.N.)

Figure S1: Infrared spectra of PHEMA in the 400-1900 cm^{-1} range (a), compared with the calculated spectra for syndiotactic (b), heterotactic (c), and isotatic (d) triads.

Figure S2: Infrared spectra of PMACC in the 400-1900 cm^{-1} range (a), compared with the calculated spectra for isotatic (b), syndiotactic (c), and heterotactic (d) triads.

Figure S3: Raman spectra of PHEMA in the 100-1900 cm^{-1} range (a), compared with the calculated spectra for syndiotactic (b), heterotactic (c), and isotatic (d) triads.

Table S1: Assignment of INS spectra of PMACC.

Table S2: Assignment of INS spectra of PHEMA.

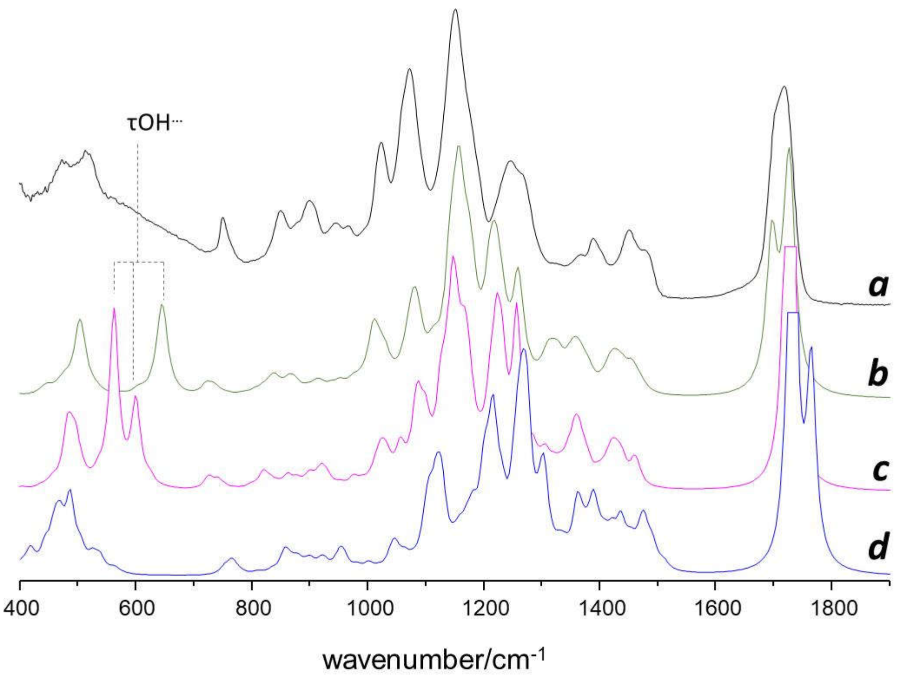

Figure S1: Infrared spectra of PHEMA in the 400-1900 cm^{-1} range (a), compared with the calculated spectra for syndiotactic (b), heterotactic (c), and isotatic (d) triads.

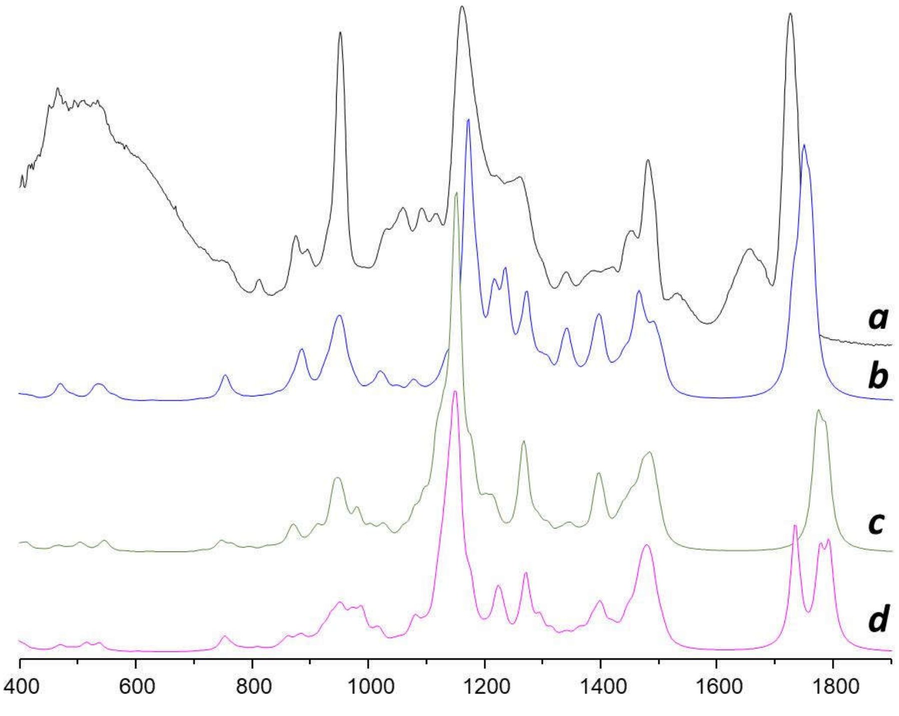

Figure S2: Infrared spectra of PMACC in the 400-1900 cm^{-1} range (a), compared with the calculated spectra for isotatic (b), syndiotactic (c), and heterotactic (d) triads.

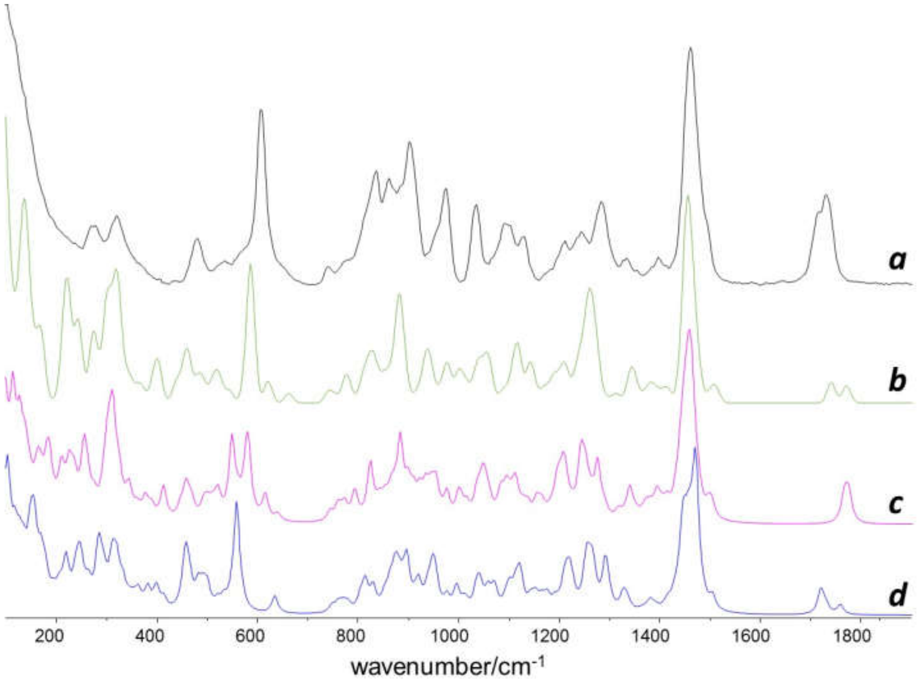

Figure S3: Raman spectra of PHEMA in the 100-1900 cm^{-1} range (a), compared with the calculated spectra for syndiotactic (b), heterotactic (c), and isotatic (d) triads.

Table S1:. Assignment of INS spectra of PMACC.

Wavenumber / cm^{-1} (average values)	Approximate Description
2995	νCH_2, νCH_3
1808	*Two-quanta transitions*
1577	$\beta sCH_3 + \beta COH$
1457-1433	$\beta s\ CH_3$, $\beta as\ CH_3$, βCH_2 scissor
1356	βCH_2 wag + βCOH
1282	βCH_2 wag + ϱCH_3
1230	βCH_2 twist, ϱCH_3
1217	βCH_2 twist, ϱCH_3
1147	$\varrho CH_3 + \nu C–O$
1080	ϱCH_3
1032	νCC (side group)
954	$\nu_{as} CN$
863	ν_{as} CN
716	νs CN (gauche)
620	$\delta CH_2–C–CH_2$ (main chain)
539	$\delta CH_2–CH_2–O$ (side group, gauche)
465	$\delta N(CH_3)_3$
450	$\delta N(CH_3)_3$
417	$\delta N(CH_3)_3$
374	$\varrho N(CH_3)_3$
326	τCH_3
292	τCH_3
268	τCH_3
144	*Deformation with CH…Cl stretch*
86	*External modes region*

Values in bold identify sharp bands or otherwise well-defined maxima. The remaining values correspond to the approximate centre of broad features or shoulders. ν = stretching; β = bending; ϱ = rocking; δ = skeletal angle deformation; τ = torsion.

Table S2: Assignment of INS spectra of PHEMA.

Wavenumber / cm^{-1}	Approximate Description
2964	νCH_2, νCH_3
1762	*Two-quanta transitions*
1453	βCH_2 scissor, βas CH_3
1380	βs CH_3 + ωCH_2 + βCOH
1280	βCH_2 twist
1238	βCH_2 twist
1105	ϱCH_2CH_2
1026	ϱCH_3
956	ϱCH_3
880	νC–C, νC–CH_3
855	CH_2 wag
765	δCH_2–C–CH_2
600	τOH···
520	δO–CH_2–CH_2 *(gauche)*
475	δO–CH_2–CH_2 *(trans)*
367	τCH_3
322	τCH_3
267	δC–O–C *out-of-plane*
221	*Deformation with OH…O stretch*
173	*Deformation with OH…O stretch*
97	*External modes region*

Values in bold identify sharp bands or otherwise well-defined maxima. The remaining values correspond to the approximate centre of broad features or shoulders. ν = stretching; β = bending; ϱ = rocking; δ = skeletal angle deformation; τ = torsion.
